# Kinematic/Dynamic SLAM for Autonomous Vehicles Using the Linear Parameter Varying Approach

**DOI:** 10.3390/s22218211

**Published:** 2022-10-26

**Authors:** Pau Vial, Vicenç Puig

**Affiliations:** Institut de Robòtica i Informàtica Industrial (CSIC-UPC), Llorens i Artigas, 4-6, 08028 Barcelona, Spain

**Keywords:** SLAM, LPV, Kalman Filter, LMI, autonomous vehicles

## Abstract

Most existing algorithms in mobile robotics consider a kinematic robot model for the the Simultaneous Localization and Mapping (SLAM) problem. However, in the case of autonomous vehicles, because of the increase in the mass and velocities, a kinematic model is not enough to characterize some physical effects as, e.g., the slip angle. For this reason, when applying SLAM to autonomous vehicles, the model used should be augmented considering both kinematic and dynamic behaviours. The inclusion of dynamic behaviour implies that nonlinearities of the vehicle model are most important. For this reason, classical observation techniques based on the the linearization of the system model around the operation point, such as the well known Extended Kalman Filter (EKF), should be improved. Consequently, new techniques of advanced control must be introduced to more efficiently treat the nonlinearities of the involved models. The Linear Parameter Varying (LPV) technique allows working with nonlinear models, making a pseudolinear representation, and establishing systematic methodologies to design state estimation schemes applying several specifications. In recent years, it has been proved in many applications that this advanced technique is very useful in real applications, and it has been already implemented in a wide variety of application fields. In this article, we present a SLAM-based localization system for an autonomous vehicle considering the dynamic behaviour using LPV techniques. Comparison results are provided to show how our proposal outperforms classical observation techniques based on model linearization.

## 1. Introduction

The European Parliament Research Service (EPRS) considers autonomous driving as one of the top ten technologies that will change people’s lives during this century [1]. The reason is that benefits provided by the autonomous driving are many and affect many social spheres. The most relevant ones are the significant reduction in the number of traffic accidents, by the elimination of the human error; the social inclusion of people with reduced mobility, by the generation of economic door-to-door services; the reduction in the traffic congestion, through the communication between vehicles and the use of an intelligent agent of the transport; and the reduction in the energetic consumption and the pollution, by the use of electric actuators and sophisticated control techniques.

To develop an autonomous vehicle, it is necessary that the vehicle software architecture includes a localization system that provides the vehicle location to the controller and the surrounding map to the trajectory planner. Then, the planner generates references and provides them to the controller to execute the vehicle control, and, finally, the localization system has to make new estimations for the carried actions. Therefore, this three modules are intimately joined, and none of them can be designed without taking the characteristics of the others into account. The authors have already largely explored the motion control [2] and the trajectory planner [3] for vehicles with evident dynamic response. Now, we are interested in extending this knowledge to build a localization node based on the solution of a Simultaneous Localization and Mapping (SLAM) problem. This problem, in the context of autonomous vehicles, has recently been reviewed in [4,5] with special emphasis on visual SLAM methods.

An important aspect to bear in mind when designing a localization system for an autonomous vehicle is its dynamic behaviour. Traditionally, SLAM algorithms have been developed for mobile platforms that move at low velocities and consequently can be modelled by only applying kinematic models [6,7]. However, for velocities larger than 5 m/s (18 km/h), kinematic models cannot describe the behaviour of a vehicle correctly enough [8]. An autonomous vehicle can very easily exceed the 5 m/s threshold during its usual operation. Therefore, the modelling of its dynamic response becomes a fundamental issue for autonomous driving. Consequently, the dynamic behaviour of the vehicle must be considered for both the control and the localization system of the autonomous vehicle.

Methods considering the dynamic model for autonomous driving of vehicles have just recently started to appear. In [9], a dynamic predictive control for the vehicle is proposed, while the localization system based on the SLAM problem is still based on a kinematic model. Although the dynamic states of the vehicle are observed, dynamics are only modelled by the integration of the accelerations of the vehicle without using a dynamic model of the vehicle, which takes into account the forces acting on it. To date, literature in this field has not yet explored SLAM for autonomous vehicles considering dynamic models. During the last decade, the development of SLAM-based techniques for autonomous driving has been focused on perception techniques and on the descriptors, especially on map management and on collaborative solutions [4]. However, all the proposed solutions still use a kinematic model for the vehicle. Therefore, it is interesting to investigate SLAM approaches for autonomous vehicles considering dynamic models.

Another relevant aspect to take into account is that vehicles are systems with an important nonlinear behaviour, especially when its dynamic response is considered [8]. Therefore, both for the observation and for the control of the vehicle, it is necessary to use nonlinear techniques. In the SLAM literature, EKF is the predominant approach (see previous referenced reviews [4,5]). UKF and CKF have also been considered as a manner to deal with the nonlinearity problem related to the SLAM for autonomous vehicles (see, e.g., [10,11]), but the the increased computational complexity is not justified by the improvement in the accuracy achieved. In terms of control, there are a few nonlinear methodologies which allow systematic tuning. The Linear Parameter Varying (LPV) technique allows working with nonlinear models and establishing systematic methodologies to tune the controller and the observer by applying various specifications using Linear Matrix Inequalities (LMI). In recent years, it has been proved that this technique of advanced control is very useful in real applications and has been implemented in a huge variety of fields [12]. There are tuning methodologies based on the LPV technique for Model Predictive Control (MPC), an optimal control methodology which is very interesting when combining the vehicle controller with the trajectory planner module. In addition, in terms of observation, LPV techniques avoid the linearization of the nonlinear problem around the operation point and allow guaranteeing the global stability of the system by keeping the Lyapunov conditions for nonlinear systems. This is the opposite of classical observation techniques, such as the Extended Kalman Filter (EKF), which only guarantee stability around the point where the linearization is calculated. Considering the coupling between the software modules, which allow the autonomous driving and the benefits provided by the LPV techniques in systems with a huge nonlinear response, it is logical to think that if the LPV technique has been applied to the vehicle controller [2], it can also be applied to the localization system. However, the LPV technique has not yet been applied to the SLAM problem. The most similar approach found in the literature is an EKF-SLAM problem solved by means fuzzy models using the Takagi–Sugeno technique and applying a purely kinematic conception [13]. It is already known that the Takagi–Sugeno technique has strong connections with LPV techniques [14]. Hence, this approach is a good reference to rewrite the SLAM problem to solve it applying LPV techniques and to incorporate the dynamic response of the vehicle, which is needed for the autonomous driving of vehicles.

In this article, a SLAM-based localization system for an autonomous vehicle in the LPV framework is proposed. The proposed approach takes into account the dynamic behaviour of the vehicle and it is adapted to the kinematic/dynamic controller architecture for an autonomous vehicle previously presented in [2]. This controller is also implemented using LPV techniques and executed into two separate layers that work with different time periods. The fastest layer is in charge of the dynamic response of the vehicle, while the slowest layer is in charge of its kinematic response. Therefore, the proposed vehicle state estimation scheme should also be organized into two separate layers. The dynamic observation must serve the dynamic controller, being part of the fastest layer. In the slowest layer, the solution to a SLAM problem should allow executing the localization task so as to serve the kinematic controller. At the same time, it should allow the map generation of the surroundings of the vehicle so as to serve as the trajectory planner module.

The main contributions with respect to the SLAM state of the art are the following:

- A SLAM LPV KF scheme is proposed that does not require the linearization of the nonlinear vehicle model but instead embeds the nonlinearities in the varying parameters.
- The proposed SLAM approach considers both the kinematic and dynamic model of the vehicle, allowing it to operate at higher speeds compared to the pure kinematic schemes available in the literature.
- A design procedure based on the LMI framework allows the offline design of the LPV KF, reducing the online computational complexity.
- The LMI framework is rooted in the Lyapunov stability theory guaranteeing the quadratic stability of the LPV KF scheme.

This article is structured as follows. Section 2 presents the kinematic and dynamic models for the autonomous vehicle considered as a case study. Section 3 describes the proposed SLAM approach formulated in the LPV framework. Section 4 shows the design procedure for the kinematic and the dynamic state estimation scheme. Section 5 presents the simulation results using the considered case study. Finally, Section 6 shows the conclusions of this work.

## 2. Problem Statement

To formulate a SLAM problem, firstly, it is necessary to define a motion model for the vehicle and an observation model for the map. The motion model defines how the vehicle moves inside the map, and the observation model allows determining how the landmarks of the map are seen from the vehicle in movement. Therefore, the motion model is a state transition function and the observation model is a kinematic transform inside the same state representation.

In this article, a two dimensional environment is considered, and, therefore, the vehicle can only do a planar movement (*x* and *y* translations and yaw rotation). Hence, ground unevenness and vehicle instabilities are not taken into account.

### 2.1. Vehicle Modelling

As already stated, mobile robotics addresses the SLAM problem using a purely kinematic model formulation. However, in the case of autonomous vehicles, because of the increase in the mass and velocities, a kinematic model is not enough to characterize some physical effects as, e.g., the slip angle. Experimental results show that during the turns of a vehicle, the linear velocity vector $\vec{v}$ detaches from the longitudinal direction of the vehicle and lateral forces appear at the wheels, which can result in drifting situations. Therefore, the motion model of the vehicle must take its kinematic and dynamic response into account.

**Figure 1:**
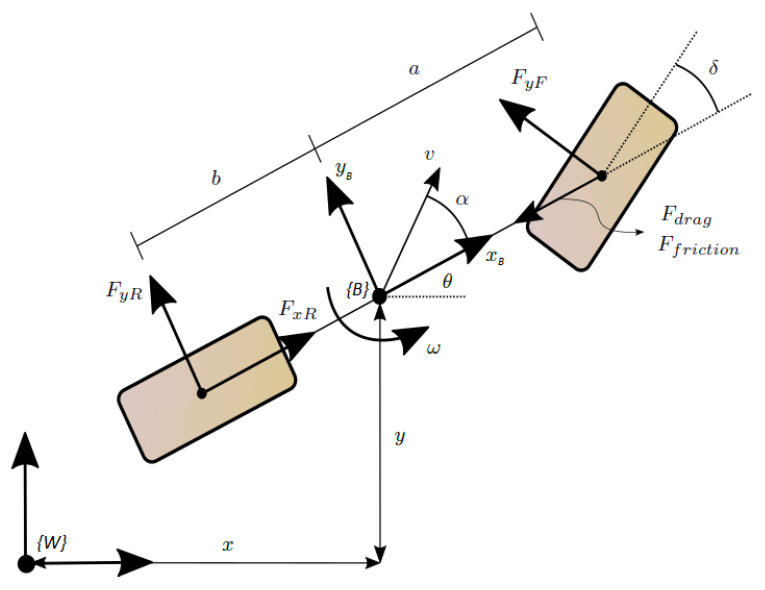
Two-wheels bicycle model used as vehicle motion model. {W} is the global world frame and {B} is the local vehicle frame.

The bicycle model proposed at [8] (see Figure 1) is taken to model the dynamic behaviour of the vehicle. Alcalá et al. [2] show that this simplified model is sufficient for dynamically controlling the vehicle and providing a good balance between representativeness and simplicity. The control actions are the traction force of the rear wheel $F_{xR}$ (accelerator and breaker) and the steering angle of the front wheel δ (steering wheel). In addition, the opposition forces acting on the vehicle are the aerodynamic force ($F_{drag}=\frac{1}{2}C_{d}\rho A{\left(v+v_{wind}\right)}^{2}$), the friction force between the vehicle and the ground ($F_{friction}=\mu mg$), and the lateral forces that appear on the wheels during the turns ($F_{yF}$ i $F_{yR}$). On the one hand, a windless environment is considered $\left(v_{wind}=0\right)$, and, therefore, the aerodynamic force is known. On the other hand, a variable friction is considered, and, therefore, the friction force acts as an unknown perturbation to the system. The dynamic states are the linear velocity *v*, the angular velocity ω, and the slip angle α; the latter appear between the linear velocity vector and the longitudinal direction of the vehicle. Applying Newton’s second law, considering a polar frame formed by the velocity vectors of the vehicle, leads to the following dynamic vehicle model

(1) $$\left\{\begin{array}{ccc} \dot{v} & = & \frac{F_{xR}\cos\alpha +F_{yF}\sin\left(\alpha -\delta \right)+F_{yR}\sin\alpha -C_{D}v^{2}}{m}-\mu g,\\ \dot{\alpha } & = & \frac{-F_{xR}\sin\alpha +F_{yF}\cos\left(\alpha -\delta \right)+F_{yR}\cos\alpha }{mv}-\omega ,\\ \dot{\omega } & = & \frac{F_{yF}a\cos\delta -F_{yR}b}{I} \end{array}\right.$$

where

$$\begin{array}{ccc} F_{yF} & = & C_{x}\left(\delta -\alpha -\frac{a\omega }{v}\right),\\ F_{yR} & = & C_{x}\left(-\alpha +\frac{b\omega }{v}\right),\\ C_{D} & = & \frac{1}{2}C_{d}\rho \;A \end{array}$$

To exemplify the results in this paper, an autonomous vehicle based on a Tazzari Zero used in the Elektra research project (http://adas.cvc.uab.es/elektra/, accessed on 24 October 2022) will be used as a case study. For this vehicle, the parameters of the dynamic model (1) are summarized in Table 1.

The kinematic model of the vehicle describes, to a fixed observer placed at the origin of the world frame {W}, how the centre of gravity of the vehicle {B} moves, giving the kinematic states *x* and *y*, corresponding to the vehicle position, and θ, corresponding to its orientation (see Figure 1). Considering the dynamic effects, the linear velocity *v* is rotated by the slip angle α, leading to the following kinematic model for the vehicle

(2) $$\left\{\begin{array}{ccc} \dot{x} & = & vs.\cos\left(\theta +\alpha \right),\\ \dot{y} & = & vs.\sin\left(\theta +\alpha \right),\\ \dot{\theta } & = & \omega . \end{array}\right.$$

Note that the slip angle α is the link between both dynamic and kinematic models.

### 2.2. Map Modelling

The observation model of the map allows performing the kinematic transformation between the observations of the surroundings of the vehicle measured from the absolute world frame {W} and the same observations measured from the mobile frame of the vehicle {V}. In this section, the observation model is derived for an exteroceptive sensor with range and bearing measures, such as a laser sensor. To do so, firstly, some considerations must be made:

- The sensor is not placed at the centre of gravity of the vehicle {V} but at a fixed point of the vehicle frame described by the pose (s,t) and the orientation β. The new frame that can be defined from this point is called the sensor frame {S}.
- To reduce the nonlinearity of the observation model, a transformation block of polar coordinates to Cartesian coordinates is installed at the sensor output:
  $$\left[\begin{array}{c} x_{l}^{s}\left(k\right)\\ y_{l}^{s}\left(k\right) \end{array}\right]=\vec{j}\left(\vec{y}\left(k\right)\right)=\left[\begin{array}{cc} \cos\left(\alpha (k)\right) & 0\\ \sin\left(\alpha (k)\right) & 0 \end{array}\right]\left[\begin{array}{c} d(k)\\ \alpha (k) \end{array}\right],$$
  where $\left(x_{l}^{s},y_{l}^{s}\right)$ is the sensor measurement Cartesian representation and (d,α) is its polar representation given by the original sensor. Using this block, it is considered that the sensor provides a Cartesian measurement to the SLAM system, as is suggested in [13].

**Figure 2:**
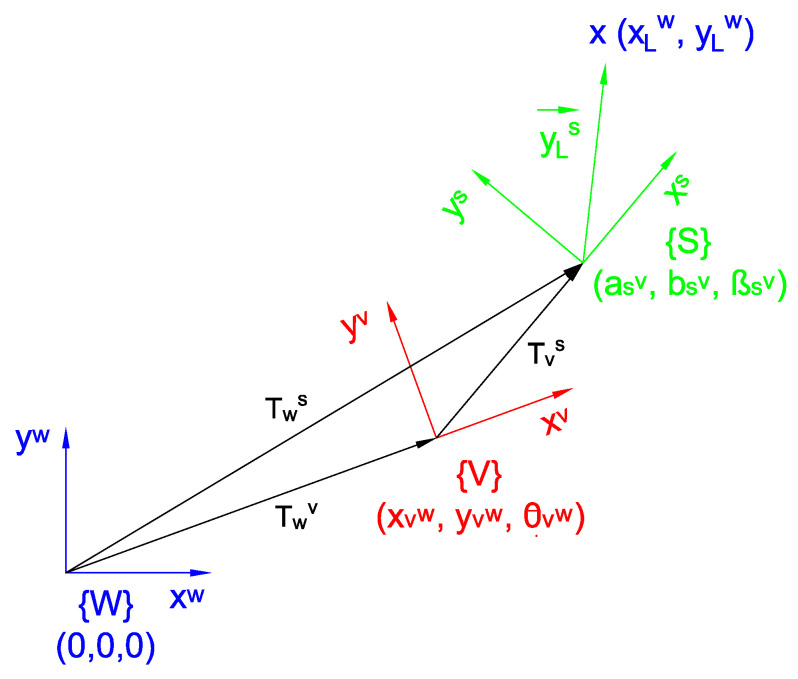
Observation model diagram. {W} is the absolute world frame, {V} is the local vehicle frame, and {S} is the local sensor frame.

According to the diagram presented in Figure 2, the observation model of the map corresponds to the geometric transform $T_{w}^{s}\left(k\right)$, which goes from the world frame {W} to the sensor frame {S}. Nevertheless, there is not enough information available to determine it completely, and it is necessary to pass by the vehicle frame {V}. Therefore, if $T_{w}^{v}\left(k\right)$ is the homogeneous transform which goes from the world frame to the vehicle frame (which is variable depending on the vehicle position) and if $T_{v}^{s}$ is the homogeneous transform which goes from the vehicle frame to the sensor frame (which is constant), then:

$$T_{w}^{s}\left(k\right)=T_{w}^{v}\left(k\right)T_{v}^{s}.$$

Considering that the vehicle is placed at the position $\left(x_{v}^{w},y_{v}^{w}\right)$ described from the world with an orientation θ and the sensor is placed at the position (s,t) described from the vehicle with an orientation β, when the pertinent transforms are applied, the analytic observation model is

(3) $$\left\{\begin{array}{ccc} x_{l}^{s}\left(k\right) & = & -x_{v}^{w}\left(k\right)\cos\left(\theta (k)+\beta \right)-y_{v}^{w}\left(k\right)\sin\left(\theta (k)+\beta \right)\\ & + & x_{l}^{w}\left(k\right)\cos\left(\theta (k)+\beta \right)+y_{l}^{w}\left(k\right)\sin\left(\theta (k)+\beta \right)+N_{1},\\ y_{l}^{s}\left(k\right) & = & x_{v}^{w}\left(k\right)\sin\left(\theta (k)+\beta \right)-y_{v}^{w}\left(k\right)\cos\left(\theta (k)+\beta \right)\\ & - & x_{l}^{w}\left(k\right)\sin\left(\theta (k)+\beta \right)+y_{l}^{w}\left(k\right)\cos\left(\theta (k)+\beta \right)+N_{2}, \end{array}\right.$$

where

$$\begin{array}{ccc} N_{1} & = & -s\cos\beta -t\sin\beta ,\\ N_{2} & = & s\sin\beta -t\cos\beta , \end{array}$$

and the installation parameters of the exteroceptive sensor are described in Table 2.

Apart from the observation model of the map, a dynamic model of the map is needed to describe the dynamic evolution of its landmarks. Since fixed landmarks at the world frame {W} are considered, modelling its performance from this frame is very easy:

(4) $$\left\{\begin{array}{ccc} {\dot{x}}_{l}^{w} & = & 0,\\ {\dot{y}}_{l}^{w} & = & 0. \end{array}\right.$$

where $\left(x_{l}^{w},y_{l}^{w}\right)$ is the position of a landmark represented in the world frame {W}.

### 2.3. Motion Model for the SLAM System

To develop a SLAM scheme using the proposed LPV approach, a linear observation model for the system is necessary. Otherwise, the complexity of the interpolation of the vertices of the polytopic LPV model grows notably. To accomplish this restriction, the map observation model must be introduced with the system motion model; thus, all system sensor measurements also become states and the observation model becomes trivial. Hence, the kinematic transform of Equation (3) must be converted into a state transition function. To do so, the time derivative of the kinematic transform is taken, and it is forced by the dynamic model of the map (Equation (4)). The following kinematic motion model for the map is obtained:

(5) $$\left\{\begin{array}{ccc} {\dot{x}}_{l}^{s} & = & -vs.\cos\left(\alpha -\beta \right)+\omega (y_{l}^{s}-N_{2}),\\ {\dot{y}}_{l}^{s} & = & -vs.\sin\left(\alpha -\beta \right)-\omega (x_{l}^{s}-N_{1}), \end{array}\right.$$

where $N_{1}$ and $N_{2}$ are defined in Equation (3), the sensor parameters are in Table 2, and $\left(x_{l}^{s},y_{l}^{s}\right)$ is the position of a landmark represented in the sensor frame {S}. This model describes how the static landmarks in the world frame {W} are moving with respect to a static observer placed at the sensor frame {S}, which introduces a robocentric point of view to the SLAM problem.

With the benefits of this derived model, now, the problem state vector includes all sensor measurements, and it is possible to write a linear observation model for the SLAM problem. Additionally, a conceptual reorganization of the problem models could be conducted as follows:

$$\begin{array}{ccc} \; & \mathrm{Vehicle}\;\mathrm{dynamic}\;\mathrm{model}\;(\mathrm{Equation}\;(1)) & \\ \; & \left(\begin{array}{ccc} vs. & \alpha & \omega \end{array}\right) & \\ \; & ↓ & \\ \; & \mathrm{Direct}\;\mathrm{observation}\;\mathrm{model}\;(\mathrm{Equation}\;(3)) & \\ \mathrm{Vehicle}\;\mathrm{kinematic} & ⟶ & \mathrm{Map}\;\mathrm{kinematic}\\ \mathrm{model}\;(\mathrm{Equation}\;(2)) & \; & \mathrm{model}\;(\mathrm{Equation}\;(5))\\ \left(\begin{array}{ccc} x_{v}^{w} & y_{v}^{w} & \theta \end{array}\right) & ⟵ & \left(\begin{array}{cc} x_{l}^{s} & y_{l}^{s} \end{array}\right)\\ \; & \mathrm{Inverse}\;\mathrm{observation}\;\mathrm{model}\;(\mathrm{Equation}\;(6)) & \\ \; & ↑ & \\ \; & \mathrm{Map}\;\mathrm{dynamic}\;\mathrm{model}\;(\mathrm{Equation}\;(4)) & \\ \; & \left(\begin{array}{cc} x_{l}^{w} & y_{l}^{w} \end{array}\right) & \end{array}$$

Note that the dynamic model defines the vehicle behaviour and provides the necessary data to the two kinematic models. These two models allow one to perform the two kinematic interpretations needed for the SLAM problem: vehicle displacement respect to the static observer at the world frame and world displacement with respect to the static observer at the vehicle frame. The direct and inverse observation models are the transforms between these two kinematic conceptions. Finally, a dynamic model for the landmarks is needed to define its evolution in the world map. However, as static landmarks at the world frame are considered, the map dynamic model is very simple, and, with the purpose of simplicity, it has been forced into the map kinematic model. Consequently, this model is not explicit in the SLAM system motion model. Hence, the static position of each landmark in the world frame is not explicit in the problem state vector and, if it is needed, it has to be obtained at each iteration applying the inverse map observation model to the state vector:

(6) $$\left\{\begin{array}{ccc} x_{l}^{w}\left(k\right) & = & x_{v}^{w}\left(k\right)+x_{l}^{s}\left(k\right)\cos\left(\theta (k)+\beta \right)-y_{l}^{s}\left(k\right)\sin\left(\theta (k)+\beta \right)+M_{1},\\ y_{l}^{w}\left(k\right) & = & y_{v}^{w}\left(k\right)+x_{l}^{s}\left(k\right)\sin\left(\theta (k)+\beta \right)+y_{l}^{s}\left(k\right)\cos\left(\theta (k)+\beta \right)+M_{2}, \end{array}\right.$$

where

$$\begin{array}{ccc} M_{1} & = & s\cos\beta -t\sin\beta ,\\ M_{2} & = & s\sin\beta +t\cos\beta ; \end{array}$$

which results from inverting Equation (3).

To summarize, the resulting motion model for the dynamic SLAM problem is:

(7) $$\left\{\begin{array}{ccc} \dot{v} & = & \frac{F_{xR}\cos\alpha +F_{yF}\sin\left(\alpha -\delta \right)+F_{yR}\sin\alpha -C_{D}v^{2}}{m}-\mu g,\\ \dot{\alpha } & = & \frac{-F_{xR}\sin\alpha +F_{yF}\cos\left(\alpha -\delta \right)+F_{yR}\cos\alpha }{mv}-\omega ,\\ \dot{\omega } & = & \frac{F_{yF}a\cos\delta -F_{yR}b}{I},\\ \dot{x_{v}^{w}} & = & vs.\cos\left(\theta +\alpha \right),\\ \dot{y_{v}^{w}} & = & vs.\sin\left(\theta +\alpha \right),\\ \dot{\theta } & = & \omega ,\\ {\dot{x}}_{l}^{s} & = & \omega y_{l}^{s}-\cos\left(\alpha -\beta \right)vs.+\left(t\cos\beta -s\sin\beta \right)\omega ,\\ {\dot{y}}_{l}^{s} & = & -\omega x_{l}^{s}-\sin\left(\alpha -\beta \right)vs.-\left(t\sin\beta +s\cos\beta \right)\omega , \end{array}\right.$$

where:

$$\begin{array}{ccc} F_{yF} & = & C_{x}\left(\delta -\alpha -\frac{a\omega }{v}\right),\\ F_{yR} & = & C_{x}\left(-\alpha +\frac{b\omega }{v}\right),\\ C_{D} & = & \frac{1}{2}C_{d}\rho \;A \end{array}$$

and its parameters are presented in Table 1 and Table 2.

## 3. Proposed Solution

**Figure 3:**
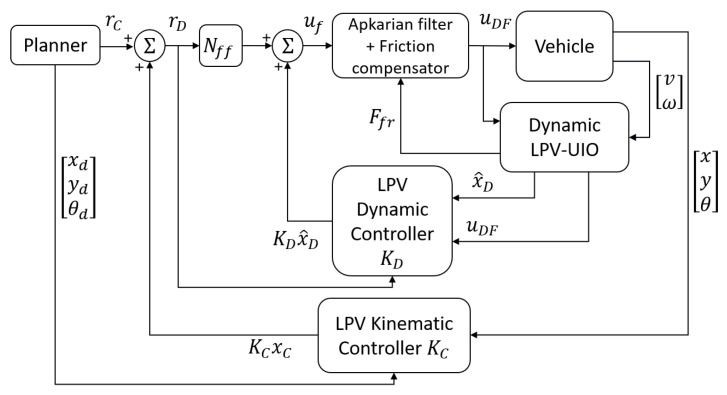
Kinematic and dynamic control architecture for an autonomous vehicle proposed in [2].

Before we introduce in detail the proposed kinematic/dynamic SLAM approach, firstly, an overall overview is presented. To develop a SLAM system that suits the kinematic/dynamic controller proposed in [2], it is necessary to structure it in layers. The fastest layer is in charge of the vehicle dynamics estimation, while the slowest layer addresses the SLAM problem using the results provided by the fastest layer (see Figure 3). Hence, the motion model (7) is divided into two submodels, one per layer. On the one hand, the state estimator of the fastest layer has to estimate the vehicle dynamic state ${\vec{x}}_{d}={\left[\begin{array}{ccc} vs. & \alpha & \omega \end{array}\right]}^{T}$ for the dynamic controller of the autonomous driving system. As inputs, it has the dynamic control signals ${\vec{u}}_{d}={\left[\begin{array}{cc} F_{xR} & \delta \end{array}\right]}^{T}$ and the unknown input $\vec{n}=\mu$, using the sensor measurements ${\vec{y}}_{d}={\left[\begin{array}{cc} vs. & \omega \end{array}\right]}^{T}$ to correct the estimation. On the other hand, the kinematic state estimator must estimate the vehicle kinematic state ${\vec{x}}_{k}={\left[\begin{array}{ccc} x_{v}^{w} & y_{v}^{w} & \theta \end{array}\right]}^{T}$ and the states of the incremental map built by the SLAM, to serve the kinematic controller and the trajectory planner system of the autonomous driving system. As input, it has the state estimation provided by the dynamic state estimator ${\vec{x}}_{d}={\left[\begin{array}{ccc} vs. & \alpha & \omega \end{array}\right]}^{T}$ that uses the sensor measurements ${\vec{y}}_{k}={\left[\begin{array}{cccccccc} x_{v}^{w} & y_{v}^{w} & \theta & x_{l1}^{s} & y_{l1}^{s} & \cdots & x_{lm}^{s} & y_{lm}^{s} \end{array}\right]}^{T}$ to correct the estimation.

Once an overview of the proposed two-layer estimation scheme is provided, in the following subsections, the detail description of each estimator is provided.

### 3.1. Dynamic State Estimation

To estimate the dynamic behaviour of the vehicle, the vehicle dynamic model (1) is considered as the motion model. This model is temporally discretized by applying the Euler approximation using the dynamic sampling time $\tau_{d}$. This allows one to obtain a pseudolinear representation applying the LPV state-space formulation by embedding the nonlinearities in the varying parameters as follows:

(8) $$\begin{array}{cc} {\vec{x}}_{d}\left(k\right) & ={\vec{f}}_{d}\left({\vec{x}}_{d}\left(k-1\right),{\vec{u}}_{d}\left(k-1\right),\vec{n}\left(k-1\right)\right)\\ & =\Phi {\left({\vec{\psi }}_{d}\right)}_{k-1}{\vec{x}}_{d}\left(k-1\right)+\Gamma {\left({\vec{\psi }}_{d}\right)}_{k-1}{\vec{u}}_{d}\left(k-1\right)+\eta \;\vec{n}\left(k-1\right) \end{array}$$

where

$$\Phi {\left({\vec{\psi }}_{d}\right)}_{k}=\left[\begin{array}{ccc} \Phi_{11} & \Phi_{12} & \Phi_{13}\\ 0 & \Phi_{22} & \Phi_{23}\\ 0 & \Phi_{32} & \Phi_{33} \end{array}\right],$$

$$\begin{array}{ccc} \Phi_{11} & = & 1-\tau_{d}\frac{1}{2}C_{d}\rho A\frac{v}{m},\\ \Phi_{12} & = & -\tau_{d}C_{x}\frac{\sin\left(\alpha -\delta \right)+\sin\alpha }{m},\\ \Phi_{13} & = & \tau_{d}C_{x}\frac{b\sin\alpha -a\sin\left(\alpha -\delta \right)}{mv},\\ \Phi_{22} & = & 1-\tau_{d}C_{x}\frac{\cos\left(\alpha -\delta \right)+\cos\alpha }{mv},\\ \Phi_{23} & = & \tau_{d}C_{x}\frac{b\cos\alpha -a\cos\left(\alpha -\delta \right)}{mv^{2}}-\tau_{d},\\ \Phi_{32} & = & \tau_{d}C_{x}\frac{b-a\cos\delta }{I},\\ \Phi_{33} & = & 1-\tau_{d}C_{x}\frac{b^{2}+a^{2}\cos\delta }{Iv}, \end{array}$$

$$\Gamma {\left({\vec{\psi }}_{d}\right)}_{k}=\left[\begin{array}{cc} \Gamma_{11} & \Gamma_{12}\\ \Gamma_{21} & \Gamma_{22}\\ 0 & \Gamma_{32} \end{array}\right],$$

$$\begin{array}{ccc} \Gamma_{11} & = & \tau_{d}\frac{\cos\alpha }{m},\\ \Gamma_{12} & = & \tau_{d}C_{x}\frac{\sin\left(\alpha -\delta \right)}{m},\\ \Gamma_{21} & = & -\tau_{d}\frac{\sin\alpha }{mv},\\ \Gamma_{22} & = & \tau_{d}C_{x}\frac{\cos\left(\alpha -\delta \right)}{mv},\\ \Gamma_{32} & = & \tau_{d}C_{x}\frac{a\cos\delta }{m}, \end{array}$$

$$\eta =\left[\begin{array}{c} -\tau_{d}g\\ 0\\ 0 \end{array}\right],$$

the model parameters are in Table 1 and the dynamic scheduling vector is ${\vec{\psi }}_{d}\left(k\right)={\left[\begin{array}{ccc} \delta (k) & v(k) & \alpha (k) \end{array}\right]}^{T}$.

If velocities *v* and ω are measured, the output equation for the dynamic system using the LPV formulation is:

(9) $${\vec{y}}_{d}\left(k\right)={\vec{h}}_{d}\left({\vec{x}}_{d}\left(k\right)\right)=C_{d}{\vec{x}}_{d}\left(k\right)$$

where

$$C_{d}=\left[\begin{array}{ccc} 1 & 0 & 0\\ 0 & 0 & 1 \end{array}\right].$$

Using these sensor measurements, the dynamic LPV system (8) is observable for all the values of the varying parameters.

The dynamic motion model (8) is additionally affected by an unknown input $\vec{n}$ that acts as a perturbation and is due to the friction force that is a function of the friction coefficient μ(k) that varies and in general is not known exactly. To be able to estimate the dynamic states without having this force, an Unknown Input Observer (UIO) scheme is proposed [16]. According to this reference, to apply the UIO, firstly, the condition rank{Cη}=rank{η} must be verified. As matrix *C* is constant and given by (9), it is easy to see that the condition is satisfied. Therefore, the UIO could be implemented. According to [16], a possible implementation is based on the transformation matrix Ω:

$$\Omega =I-\eta \left[\;{\left(C\eta \right)}^{T}C\eta \right]{\;}^{-1}{\left(C\eta \right)}^{T}C=\left[\begin{array}{ccc} 0 & 0 & 0\\ 0 & 1 & 0\\ 0 & 0 & 1 \end{array}\right].$$

which allows one to produce a new motion model decoupled from the perturbation and can be estimated using the input/output measurements:

(10) $$\begin{array}{cc} {\vec{x}}_{d}\left(k\right) & ={\vec{f}}_{d}\left({\vec{x}}_{d}\left(k-1\right),{\vec{u}}_{d}\left(k-1\right),{\vec{y}}_{d}\left(k\right)\right)\\ & =\bar{\Phi }{\left({\vec{\psi }}_{d}\right)}_{k-1}{\vec{x}}_{d}\left(k-1\right)+\bar{\Gamma }{\left({\vec{\psi }}_{d}\right)}_{k-1}{\vec{u}}_{d}\left(k-1\right)+\Sigma \;{\vec{y}}_{d}\left(k\right) \end{array}$$

where

$$\begin{array}{ccc} \bar{\Phi }{\left(\vec{\psi_{d}}\right)}_{k} & = & \Omega \Phi {\left({\vec{\psi }}_{d}\right)}_{k},\\ \bar{\Gamma }{\left(\vec{\psi_{d}}\right)}_{k} & = & \Omega \Gamma {\left({\vec{\psi }}_{d}\right)}_{k},\\ \Sigma & = & \eta \left[\;{\left(C\eta \right)}^{T}C\eta \right]{\;}^{-1}{\left(C\eta \right)}^{T}, \end{array}$$

Note that all the involved matrices are defined in Equation (8).

### 3.2. Kinematic State Estimation and SLAM

To solve the SLAM problem, the motion and observation models of the kinematic layer must be defined. The motion model includes the vehicle kinematic model (2), the map kinematic model, and the implicit map dynamic model. As in the case of the dynamic model, they are temporally discretized by applying the Euler approximation using the kinematic sampling time $\tau_{k}$ and expressed in pseudolinear representation using the state-space LPV representation based on nonlinear embedding the nonlinearities in the varying parameters. In the case of considering *n* landmarks, the obtained LPV representation is:

(11) $${\vec{x}}_{k}\left(k\right)={\vec{f}}_{k}\left({\vec{x}}_{k}\left(k-1\right),{\vec{x}}_{d}\left(k-1\right)\right)=\Phi {\left({\vec{\psi }}_{k}\right)}_{k-1}{\vec{x}}_{k}\left(k-1\right)+\Gamma {\left({\vec{\psi }}_{k}\right)}_{k-1}{\vec{x}}_{d}\left(k-1\right),$$

where

$$\Phi {\left({\vec{\psi }}_{k}\right)}_{k}=\left[\begin{array}{cccccccc} 1 & 0 & 0 & 0 & 0 & \cdots & 0 & 0\\ 0 & 1 & 0 & 0 & 0 & \cdots & 0 & 0\\ 0 & 0 & 1 & 0 & 0 & \cdots & 0 & 0\\ 0 & 0 & 0 & 1 & \phi & \cdots & 0 & 0\\ 0 & 0 & 0 & -\phi & 1 & \cdots & 0 & 0\\ \vdots & \vdots & \vdots & \vdots & \vdots & \ddots & \vdots & \vdots \\ 0 & 0 & 0 & 0 & 0 & \cdots & 1 & \phi \\ 0 & 0 & 0 & 0 & 0 & \cdots & -\phi & 1 \end{array}\right],$$

$$\begin{array}{ccc} \phi & = & \tau_{k}\omega , \end{array}$$

$$\Gamma {\left({\vec{\psi }}_{k}\right)}_{k}=\left[\begin{array}{ccc} \Gamma_{11} & 0 & 0\\ \Gamma_{21} & 0 & 0\\ 0 & 0 & \Gamma_{33}\\ \Gamma_{41} & 0 & \Gamma_{43}\\ \Gamma_{51} & 0 & \Gamma_{53}\\ \vdots & \vdots & \vdots \\ \Gamma_{41} & 0 & \Gamma_{43}\\ \Gamma_{51} & 0 & \Gamma_{53} \end{array}\right],$$

$$\begin{array}{ccc} \Gamma_{11} & = & \tau_{k}\cos\left(\theta +\alpha \right),\\ \Gamma_{21} & = & \tau_{k}\sin\left(\theta +\alpha \right),\\ \Gamma_{33} & = & \tau_{k},\\ \Gamma_{41} & = & -\tau_{k}\cos\left(\alpha -\beta \right),\\ \Gamma_{43} & = & \tau_{k}\left(t\cos\beta -s\sin\beta \right),\\ \Gamma_{51} & = & -\tau_{k}\sin\left(\alpha -\beta \right),\\ \Gamma_{53} & = & -\tau_{k}\left(t\sin\beta +s\cos\beta \right), \end{array}$$

where the model parameters are summarized in Table 2 and the kinematic scheduling vector is ${\vec{\psi }}_{k}\left(k\right)=\left[\begin{array}{ccc} \alpha (k) & \omega (k) & \theta (k) \end{array}\right]$. Matrix $\Phi {\left({\vec{\psi }}_{k}\right)}_{k}$ is square with dimension 3+2n and the dimensions of matrix $\Gamma {\left({\vec{\psi }}_{k}\right)}_{k}$ are (3+2n)×3.

To solve the SLAM problem, it is necessary to bear in mind that the kinematic state vector is formed by the *n* discovered landmarks which form the incremental map. Note that technically it is only possible to consider a subset of the discovered landmarks, called the active landmarks. From that subset of active landmarks, a group of measurements are correlated with the known landmarks of the map, which are used to perform the kinematic observation task. The rest of active landmarks are new discoveries and will be included in the map once the kinematic observation is performed. Hence, the measurements of the kinematic system are the pose of the vehicle in the world frame and the position of the *m* correlated active landmarks measured in the sensor frame are

$${\vec{y}}_{k}={\left[\begin{array}{cccccccc} x_{v}^{w}\left(k\right) & y_{v}^{w}\left(k\right) & \theta & x_{l1}^{s}\left(k\right) & y_{l1}^{s} & \cdots & x_{lm}^{s}\left(k\right) & y_{lm}^{s}\left(k\right) \end{array}\right]}^{T}.$$

The resulting observation model is

(12) $${\vec{y}}_{k}\left(k\right)={\vec{h}}_{k}\left({\vec{x}}_{k}\left(k\right)\right)=C_{k}{\vec{x}}_{k}\left(k\right)$$

where

$$C_{k}=\left[\begin{array}{cccccccc} 1 & 0 & 0 & 0 & 0 & \cdots & 0 & 0\\ 0 & 1 & 0 & 0 & 0 & \cdots & 0 & 0\\ 0 & 0 & 1 & 0 & 0 & \cdots & 0 & 0\\ 0 & 0 & 0 & 1 & 0 & \cdots & 0 & 0\\ 0 & 0 & 0 & 0 & 1 & \cdots & 0 & 0\\ \vdots & \vdots & \vdots & \vdots & \vdots & \ddots & \vdots & \vdots \\ 0 & 0 & 0 & 0 & 0 & \cdots & 1 & 0\\ 0 & 0 & 0 & 0 & 0 & \cdots & 0 & 1 \end{array}\right]$$

with dimensions (3+2m)×(3+2n).

$${\vec{\hat{x}}}_{k}\left(k\right)={\vec{\hat{x}}}_{k}^{+}\left(k\right)+L_{k}\left(k\right)\left[{\vec{y}}_{k}\left(k\right)-C_{k}{\vec{\hat{x}}}_{k}^{+}\left(k\right)\right]$$

### 3.3. State Estimation Scheme

Once the models used for state estimation and SLAM have been introduced, they are used through the following estimation structure,

(13) $${\vec{\hat{x}}}_{d}\left(k\right)={\vec{\hat{x}}}_{d}^{+}\left(k\right)+L_{d}\left(k\right)\left[{\vec{y}}_{d}\left(k\right)-C_{d}\left(k\right){\vec{\hat{x}}}_{d}^{+}\left(k\right)\right]$$

where $C_{d}$ is the output matrix defined in Equation (9) in the case of the dynamic estimation or in Equation (12) in the case of SLAM. The difference between the different proposed estimators is the considered model described in previous subsections and the way the correcting gain $L_{d}\left(k\right)$ is obtained using the design procedures presented in the next section.

Using (13) at every time iteration *k*, the vehicle motion model must be evaluated using the control signals ${\vec{u}}_{d}\left(k-1\right)$ and previous state estimations ${\vec{\hat{x}}}_{d}^{+}\left(k\right)$. The sensor measurements ${\vec{y}}_{d}\left(k\right)$ are used to correct the state estimations provided by the model through the correcting factor $L_{d}\left(k\right)$.

## 4. Design Procedures

In this section, two different procedures are proposed to determine the correction gain $L_{d}\left(k\right)$ required for the state estimation scheme (13). The first approach is based on solving online the Ricatti equation as in EKF [17]. However, the proposed approach avoids the linearization of the model required in EKF by using the pseudolinearization offered by the LPV formulation of the model presented in the previous section for the different models considered. The second approach is based on using the polytopic representation of the LPV model. This approach allows solving the Ricatti equations formulated as LMIs [17] offline only in the vertices of the polytopic model. During the online operation, the value of correction gain $L_{d}\left(k\right)$ at each operation point is calculated online by interpolation of the solution found offline at the vertices of the polytopic LPV model [12].

### 4.1. Online Approach

To apply the online approach, the matrices of the pseudolinear system model representation are evaluated at the current operating point. The calculation of the gain $L_{d}\left(k\right)$ is based on solving the Ricatti Equation of the Kalman Filter using the current value of system matrices. This implies performing the following calculations at each time iteration *k* to determine the gain $L_{d}\left(k\right)$ and the covariance matrix $P_{d}\left(k\right)$

$$\begin{array}{ccc} P_{d}^{+}\left(k\right) & = & \bar{\Phi }{\left({\vec{\psi }}_{d}\right)}_{k-1}P_{d}\left(k-1\right)\bar{\Phi }{\left({\vec{\psi }}_{d}\right)}_{k-1}^{T}+\bar{\Gamma }{\left({\vec{\psi }}_{d}\right)}_{k-1}Q_{d}\bar{\Gamma }{\left({\vec{\psi }}_{d}\right)}_{k-1}^{T}+\Sigma R_{d}\Sigma ,\\ Z(k) & = & C_{d}P_{d}^{+}\left(k\right)C_{d}^{T}+R_{d},\\ L_{d}\left(k\right) & = & P_{d}^{+}\left(k\right)C_{d}^{T}Z{\left(k\right)}^{-1},\\ P_{d}\left(k\right) & = & P_{d}^{+}\left(k\right)-L_{d}\left(k\right)C_{d}^{T}P_{d}^{+}\left(k\right). \end{array}$$

Matrices $\bar{\Phi }{\left({\vec{\psi }}_{d}\right)}_{k-1}$, $\bar{\Phi }{\left({\vec{\psi }}_{d}\right)}_{k-1}$ and Σ are defined in Equation (10), matrix $C_{d}$ is defined in Equation (9), and $Q_{d}$ and $R_{d}$ are, respectively, the covariance matrices of the disturbances and sensor measurements.

In the case of SLAM, the following calculation must be performed at each iteration to determine the gain $L_{k}\left(k\right)$ and the covariance matrix $P_{k}\left(k\right)$:

$$\begin{array}{ccc} P_{k}^{+}\left(k\right) & = & \Phi {\left({\vec{\psi }}_{k}\right)}_{k-1}P_{k}\left(k-1\right)\Phi {\left({\vec{\psi }}_{k}\right)}_{k-1}^{T}+\Gamma {\left({\vec{\psi }}_{k}\right)}_{k-1}P_{d}\left(k-1\right)\Gamma {\left({\vec{\psi }}_{k}\right)}_{k-1}^{T},\\ Z(k) & = & C_{k}P_{k}^{+}\left[rl,rl\right]\left(k\right)C_{k}^{T}+R_{k},\\ L_{k}\left(k\right) & = & P_{k}^{+}\left[rm,rl\right]\left(k\right)C_{k}^{T}Z{\left(k\right)}^{-1},\\ P_{k}\left(k\right) & = & P_{k}^{+}\left[rm,rm\right]\left(k\right)-L_{k}\left(k\right)C_{k}^{T}P_{k}^{+}\left[rl,rm\right]\left(k\right). \end{array}$$

Matrices $\Phi {\left({\vec{\psi }}_{k}\right)}_{k-1}$ and $\Gamma {\left({\vec{\psi }}_{k}\right)}_{k-1}$ are defined in Equation (11), and matrix $C_{k}$ is defined in Equation (12). $P_{d}\left(k-1\right)$ is the covariance matrix of the dynamic estimation applied to the kinematic estimation. In contrast, $P_{k}\left(k-1\right)$ is the variance matrix of the kinematic estimation carried out on the previous iteration. As seen, both layers are linked by the variance matrix $P_{d}$. Finally, rl refers to the dimensions of matrix $P_{k}^{+}\left(k\right)$ corresponding to the vehicle kinematics and to the active landmarks, and rm refers to all dimensions of matrix $P_{k}^{+}\left(k\right)$.

### 4.2. Offline Approach

The offline approach is based on the use of the polytopic LPV vehicle model.

To define the polytopic LPV vehicle model, firstly, it must be noted that the dimension of the dynamic scheduling vector ${\vec{\psi }}_{d}$ is $n_{\psi }=3$. Hence, the dynamic polytopic model has $n_{\Psi }=8$ vertices ${\vec{\psi }}_{di}$, which are:

$$\Psi_{d}=\left[\begin{array}{ccc} \underline{\delta } & \underline{v} & \underline{\alpha }\\ \underline{\delta } & \underline{v} & \bar{\alpha }\\ \underline{\delta } & \bar{v} & \underline{\alpha }\\ \underline{\delta } & \bar{v} & \bar{\alpha }\\ \bar{\delta } & \underline{v} & \underline{\alpha }\\ \bar{\delta } & \underline{v} & \bar{\alpha }\\ \bar{\delta } & \bar{v} & \underline{\alpha }\\ \bar{\delta } & \bar{v} & \bar{\alpha } \end{array}\right],$$

where the underlined and the overlined represent, respectively, the lowest and the highest limit for each scheduling variable.

Then, the following Kalman Filter Ricatti equation expressed in LMI form [17] must be solved for each vertex of the polytopic LPV model:

(14) $$\begin{array}{c} \left[\begin{array}{cccc} -Y_{d} & Y_{d}\bar{\Phi }\left({\vec{\psi }}_{di}\right)-W\left({\vec{\psi }}_{di}\right)C_{d} & Y_{d}\bar{Q}{\left({\vec{\psi }}_{di}\right)}^{T} & W({\vec{\psi }}_{di})\\ \bar{\Phi }{\left({\vec{\psi }}_{di}\right)}^{T}Y_{d}-C_{d}^{T}W{\left({\vec{\psi }}_{di}\right)}^{T} & -Y_{d} & 0 & 0\\ \bar{Q}\left({\vec{\psi }}_{di}\right)Y_{d} & 0 & -I & 0\\ W{\left({\vec{\psi }}_{di}\right)}^{T} & 0 & 0 & -R^{-1} \end{array}\right]\leq 0\;for\;i=1\cdots n_{\Psi } \end{array}$$

where

$$\begin{array}{ccc} \bar{Q}\left({\vec{\psi }}_{di}\right) & = & \sqrt{\bar{\Gamma }\left({\vec{\psi }}_{di}\right)Q_{d}\bar{\Gamma }{\left({\vec{\psi }}_{di}\right)}^{T}+\Sigma R_{d}\Sigma^{T}},\\ W({\vec{\psi }}_{di}) & = & Y_{d}^{-1}L_{d}\left({\vec{\psi }}_{di}\right). \end{array}$$

$\bar{\Phi }\left({\vec{\psi }}_{di}\right)$, $\bar{\Gamma }\left({\vec{\psi }}_{di}\right)$ and Σ are defined in Equation (10), and $C_{d}$ is the output matrix defined in Equation (9). $Q_{d}$ and $R_{d}$ are the variance matrices of the control signals and sensor measurements. An additional LMI has to be included to guarantee the stability in the Lyapunov sense of the resulting polytopic LPV estimation scheme:

(15) $$\left[\begin{array}{cc} \gamma I & I\\ I & Y_{d} \end{array}\right]\geq 0,$$

where *I* is a three-dimensional identity matrix.

Finally, an optimization problem considering the previous LMIs (14) and (15) as constraints must be solved, where the minimization goal is the scalar γ and the decision variables are matrix $Y_{d}=P_{d}^{-1}$ and matrices $W({\vec{\psi }}_{di})$. The problem solution provides the gain $L_{d}\left({\vec{\psi }}_{di}\right)$ for each vertex of the dynamic polytopic LPV model.

Once the gains $L_{d}\left({\vec{\psi }}_{di}\right)$ for each vertex of the polytopic model are obtained offline, the gain $L_{d}\left({\vec{\psi }}_{d}\right)\left(k\right)$ is adapted in function of the operation point as follows

(16) $$L_{d}\left({\vec{\psi }}_{d}\right)\left(k\right)=\sum_{i=1}^{n_{\Psi }}\mu_{di}\left(k\right)L_{d}\left({\vec{\psi }}_{di}\right).$$

where the interpolation factors $\mu_{di}\left(k\right)$ for each vertex of the polytopic model are obtained as follows:

(17) $$\mu_{di}\left(k\right)=\prod_{j=1}^{n_{\psi }}\varepsilon_{ij}\left(\eta_{0}^{j}\left(k\right),\eta_{1}^{j}\left(k\right)\right)\;for\;i=1\ldots n_{\Psi }$$

with

(18) $$\begin{array}{cc} \begin{array}{ccc} \eta_{0}^{j}\left(k\right) & = & \frac{\bar{\psi_{j}}-\psi_{j}\left(k\right)}{\bar{\psi_{j}}-\underline{\psi_{j}}},\\ \eta_{1}^{j}\left(k\right) & = & 1-\eta_{0}^{j}\left(k\right), \end{array} & \;for\;j=1\ldots n_{\psi }, \end{array}$$

taking into account the limits for each scheduling variable $\psi_{j}\left(k\right)$.

Using the LPV polytopic approach to solve a SLAM problem requires limiting the dimension of the map state vector of the kinematic system to only the *m* active landmarks correlated with the known map. In this way, the dimension of the state vector does not grow indefinitely as the problem advances and, by fixing a limit ρ to the maximum number of correlated active landmarks at each iteration, the possible ρ+1 dimensions for the state vector can be forecast in advance. As the kinematic scheduling vector is not affected by the position of any landmark, the polytopic model generated for the subset of *m* correlated active landmarks is valid for any subset of active landmarks, while the dimension of the subset remains at *m*. Following this approach, it is possible to tune ρ+1 different gains offline. Furthermore, during the observation algorithm, it is possible to choose the convenient set of gains depending on the *m* correlated active landmarks available at each operation point. Unlike with the online Ricatti methodology, the SLAM problem solved by means of the polytopic approach uses a limited memory considering only the landmarks close to the surroundings of the vehicle to perform the localization task, without doing corrections to the entirety of the incremental map built during the whole execution. Nevertheless, this problem relaxation allows the observability condition of the kinematic layer to be accomplished.

To define the polytopic model of the kinematic system, firstly, it must be noted that the dimension of the kinematic scheduling vector ${\vec{\psi }}_{k}$ is $n_{\psi }=3$. Hence, the kinematic polytopic model has $n_{\Psi }=8$ vertices ${\vec{\psi }}_{ki}$ defined as follows:

$$\Psi_{k}=\left[\begin{array}{ccc} \underline{\alpha } & \underline{\omega } & \underline{\theta }\\ \underline{\alpha } & \underline{\omega } & \bar{\theta }\\ \underline{\alpha } & \bar{\omega } & \underline{\theta }\\ \underline{\alpha } & \bar{\omega } & \bar{\theta }\\ \bar{\alpha } & \underline{\omega } & \underline{\theta }\\ \bar{\alpha } & \underline{\omega } & \bar{\theta }\\ \bar{\alpha } & \bar{\omega } & \underline{\theta }\\ \bar{\alpha } & \bar{\omega } & \bar{\theta } \end{array}\right],$$

where the underlined and the overlined represent, respectively, the lowest and the highest limit for each scheduling variable.

The dimension of the kinematic system is variable because of the number of landmarks considered in the SLAM problem. Therefore, if ϵ∈[0,ρ] is the number of active landmarks correlated with the known map for an operation point of the observer system, then a tuning process must be performed for each possible dimension ϵ for the correlated active landmarks subset. Hence, ρ+1 different sets of gains must be calculated, because the case without any measured known landmarks should be taken into account. To tune the set of gains for ϵ landmarks, the LMI for the optimum gain [17] has to be considered for each vertex of the polytopic model:

$$\begin{array}{c} \left[\begin{array}{cccc} -Y_{k\epsilon } & Y_{k\epsilon }\Phi_{\epsilon }\left({\vec{\psi }}_{ki}\right)-W_{\epsilon }\left({\vec{\psi }}_{ki}\right)C_{k\epsilon } & Y_{k\epsilon }\bar{Q}{\left({\vec{\psi }}_{ki}\right)}^{T} & W_{\epsilon }\left({\vec{\psi }}_{ki}\right)\\ \Phi_{\epsilon }{\left({\vec{\psi }}_{ki}\right)}^{T}Y_{k\epsilon }-C_{k\epsilon }^{T}W_{\epsilon }{\left({\vec{\psi }}_{ki}\right)}^{T} & -Y_{k\epsilon } & 0 & 0\\ \bar{Q}\left({\vec{\psi }}_{ki}\right)Y_{k\epsilon } & 0 & -I & 0\\ W_{\epsilon }{\left({\vec{\psi }}_{ki}\right)}^{T} & 0 & 0 & -R_{k\epsilon }^{-1} \end{array}\right]\leq 0\;for\;i=1\ldots n_{\Psi } \end{array}$$

where

$$\begin{array}{ccc} \bar{Q}\left({\vec{\psi }}_{ki}\right) & = & \sqrt{\Gamma_{\epsilon }\left({\vec{\psi }}_{ki}\right)P_{d}\Gamma_{\epsilon }{\left({\vec{\psi }}_{ki}\right)}^{T}},\\ W_{\epsilon }\left({\vec{\psi }}_{ki}\right) & = & Y_{k\epsilon }^{-1}L_{\epsilon }\left({\vec{\psi }}_{ki}\right). \end{array}$$

$\Phi_{\epsilon }\left({\vec{\psi }}_{ki}\right)$ and $\Gamma_{\epsilon }\left({\vec{\psi }}_{ki}\right)$ are defined in Equation (11), $C_{k\epsilon }$ is the output matrix defined in Equation (12), and $R_{k}$ and $P_{d}$ are, respectively, the covariance matrices of the sensor measurements and the dynamic estimations used. As can be seen, all matrices are defined according to the fixed ϵ active landmarks, and the covariance matrix $P_{d}$ is the link between the kinematic/dynamic estimation layers. Next, an additional LMI must be included to guarantee the global stability of the polytopic estimation scheme to accomplish Lyapunov conditions:

$$H=\left[\begin{array}{cc} \gamma I & I\\ I & Y_{k\epsilon } \end{array}\right]\geq 0,$$

where *I* is an n+2ϵ dimensional identity matrix.

Finally, the optimization problem defined by the resulting LMIs must be solved, where the minimization goal is the scalar γ and the decision variables are the matrix $Y_{k\epsilon }$ and the matrices $W_{\epsilon }\left({\vec{\psi }}_{ki}\right)$. The problem solution provides a gain $L_{\epsilon }\left({\vec{\psi }}_{ki}\right)$ for each vertex of the kinematic polytopic model. This set of gains for the subset of ϵ active landmarks must be saved in the vehicle estimator memory because it must be used during the estimation process every time ϵ correlated active landmarks are available. The described process must be repeated for each ρ+1 possible subset of active landmarks.

To determine the polytopic gain $L_{\epsilon }\left({\vec{\psi }}_{ki}\right)\left(k\right)$ for each operation point inside the estimation loop, firstly, the ϵ active landmarks correlated with the map must be determined, that is, for which map landmarks are the vehicle sensors able to obtain measurements at the current iteration. This information allows fixing the system state ${\vec{x}}_{k}\left[rl\right]\left(k\right)$ for the operation point. Next, the set of gains $L_{\epsilon }\left({\vec{\psi }}_{ki}\right)$ for ϵ active landmarks has to be searched in the vehicle estimator memory. Afterwards, the direct and inverse unit scaling of each scheduling variable must be determined according to the previous kinematic estimations ${\vec{x}}_{k}\left(k-1\right)$, applying Equation (18) for each scheduling variable. Then, the interpolation factors $\mu_{ki}\left(k\right)$ for each vertex of the polytopic estimator must be determined by applying Equation (17) and following the logic used to define $\Psi_{k}$. Lastly, the interpolated gain must be calculated by applying Equation (16) and the interpolation factors $\mu_{ki}\left(k\right)$ previously found.

## 5. Simulation Results

In this section, simulation results are provided to illustrate the performance of the proposed approaches and compare the differences between the two design procedures presented in Section 4. Furthermore, a comparison with the classical EKF is provided to prove the interest of the introduced techniques.

### 5.1. Simulation Set-Up

To conduct these simulations, a handmade Matlab environment inspired by the SLAM Toolbox for Matlab [18] is used, and the control data are taken from the experimentation carried in a previous work [2]. As a world map, a synthetic map is taken, formed by a mesh of 12×40 elements uniformly distributed between coordinates −50 m and 1050 m in the *x* direction and between −50 m and 450 m in the *y* direction. Hence, the vehicle has 480 available landmarks along its path, although a limit of ρ=10 active landmarks at each iteration is set. Simulations are conducted considering the Tazzari Zero autonomous vehicle, whose parameters are shown in Table 1, as well as the exteroceptive sensor installation parameters shown in Table 2. The limits for the scheduling variables of the kinematic/dynamic LPV models are set as follows:

$$\begin{array}{cccccc} \underline{\delta } & = & -25^{\circ }, & \bar{\delta } & = & 25^{\circ },\\ \underline{v} & = & 2\;m/s, & \bar{v} & = & 18\;m/s,\\ \underline{\alpha } & = & -0.1\;\mathrm{rad}, & \bar{\alpha } & = & 0.1\;\mathrm{rad},\\ \underline{\theta } & = & -90^{\circ }, & \bar{\theta } & = & 160^{\circ },\\ \underline{\omega } & = & -0.2\;\mathrm{rad}/s, & \bar{\omega } & = & 0.2\;\mathrm{rad}/s. \end{array}$$

A sampling time of $\tau_{d}=1$ ms is used for the dynamic state estimation layer, while a sampling time of $\tau_{k}=100$ ms is considered for the SLAM layer. For the design of the dynamic state estimator, diagonal covariance matrices $Q_{d}=\left[\begin{array}{cc} 1, & 1\times 10^{-4} \end{array}\right]$, and $R_{d}=\left[\begin{array}{cc} 1\times 10^{-2}, & 1\times 10^{-4} \end{array}\right]$ are considered. For the SLAM layer, diagonal covariance matrices $R_{k}=\left[\begin{array}{ccc} 1\times 10^{-2}, & 1\times 10^{-2}, & 1\times 10^{-6} \end{array}\right]$, and $R_{m}=\left[\begin{array}{cc} 1\times 10^{-2}, & 1\times 10^{-2} \end{array}\right]$ are used, where $R_{k}$ is related to the kinematic sensors of the vehicle and $R_{m}$ is related to the measure of a single landmark. To design the polytopic LPV estimators following the offline approach presented in Section 4, Mosek solver [19] is used to solve all the involved optimization problems.

### 5.2. Simulation Scenarios

**Figure 4:**
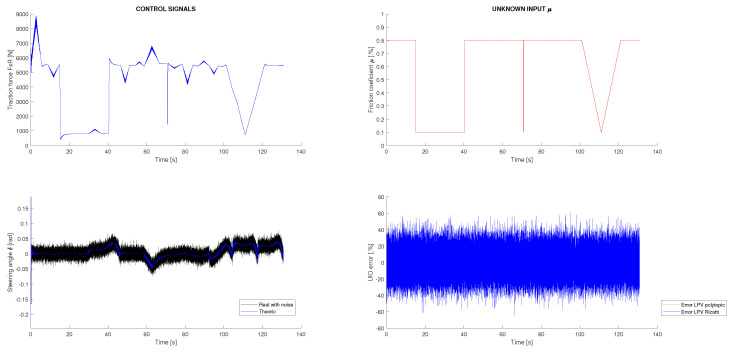
Vehicle dynamic inputs in the simulation with friction and Gaussian noise.

**Figure 5:**
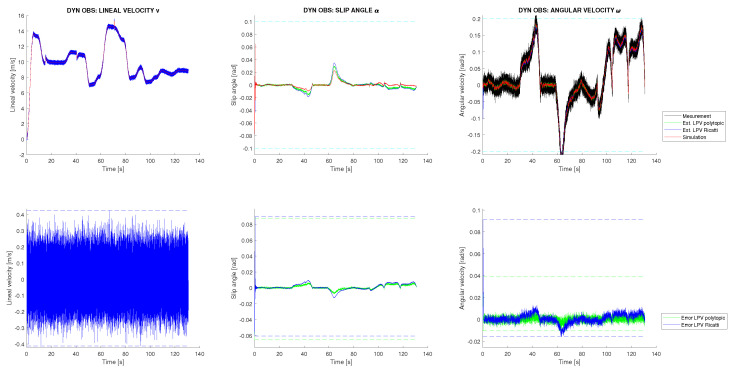
Vehicle dynamic state estimation from the simulation with friction and Gaussian noise/disturbances.

Two different experiments are conducted. In both cases, a variable friction between the vehicle and the ground is considered, as well as additive perturbations and sensor noise. All perturbations and noises are assumed to be Gaussian and are generated according to their corresponding covariance matrices *Q* and *R*, which were previously defined. In Figure 4, top right, the variable friction applied to the system is shown, and in Figure 4 left, the dynamic control signals ${\vec{u}}_{d}$ with additive Gaussian disturbances are presented. Using these data as inputs to the vehicle simulator and the sensor measurements ${\vec{y}}_{d}$ obtained, the dynamic response of the vehicle could be estimated. In both simulation scenarios, these inputs are common, and consequently, the dynamic estimation results are the same. These results are shown in Figure 5, where it can be observed that both estimation techniques provide good results with a very similar performance. Both techniques filter the system disturbances, although the polytopic LPV approach presents less estimation error accumulation. As will be shown next, this result will be amplified in the kinematic SLAM layer. Furthermore, in Figure 5 it is shown that for state α, which is not measured, a good estimation is achieved. In contrast to all of that, in Figure 4, right, the friction estimation performed by the UIO is shown. It can be seen that this estimation is equivalent for both estimation techniques, although the UIO associates most of the disturbances received with the friction, complicating the distinction between process disturbances and friction force. Furthermore, the application of an UIO to this specific problem implies that the measurement of the state *v* is analytically assigned directly to the estimation, blocking any correction or filtering process to that state. This phenomenon is shown in Figure 5, where it is seen how the estimation of the state *v* could not be filtered. In conclusion, although the UIO is necessary to compensate the variable perturbation introduced by the vehicle friction, it cannot completely eliminate the effect of additive Gaussian disturbances.

**Figure 6:**
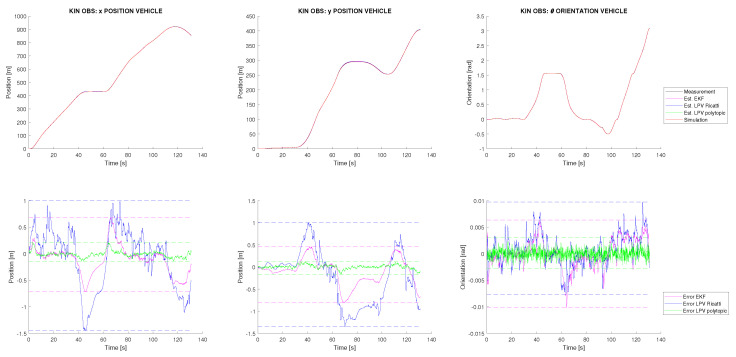
Vehicle kinematic state estimation from the simulation with friction, Gaussian noise, and noisy initialization of landmarks.

Using the dynamic estimations, the kinematic SLAM estimation can be performed. Two experiments are proposed to evaluate the robustness of the proposed SLAM approach. In the first experiment, initialization of landmarks is corrupted with an additive Gaussian noise with a standard deviation of 10 m, which allows modelling an exteroceptive sensor with a very bad state initialization. The results in this case are shown in Figure 6, where significant differences are observed between the online and offline design approaches. For this reason, the classical solution using an EKF, based on first-order linearization, is also plotted and used as a reference for comparison. On the one hand, it can be observed that the online approach presents significant biases, following the same trend as the EKF but accumulating more error. On the other hand, the offline design approach based on the polytopic LPV estimator accumulates less error than the EKF and provides a vehicle localization with an order accuracy of a few decimeters for the position, one order of magnitude less than the estimation performed by the Ricatti observer. These error accumulations appear during the vehicle turns, when the $\vec{v}$ direction is detached from the longitudinal direction of the vehicle, dynamic effects become evident and drifts can appear at the vehicle wheels. As the dynamic behaviour is affected by important nonlinearities, these results show how the polytopic observer is able to deal with the nonlinearities problem, and therefore, it becomes the most dynamically sensitive, improving the classical EKF. Despite this, note that the estimation for the polytopic observer is a bit more noisy than the EKF, especially for the vehicle orientation. However, the global accuracy improves with respect to the EKF, and it can be concluded that the polytopic observer stands out for the localization task.

**Figure 7:**
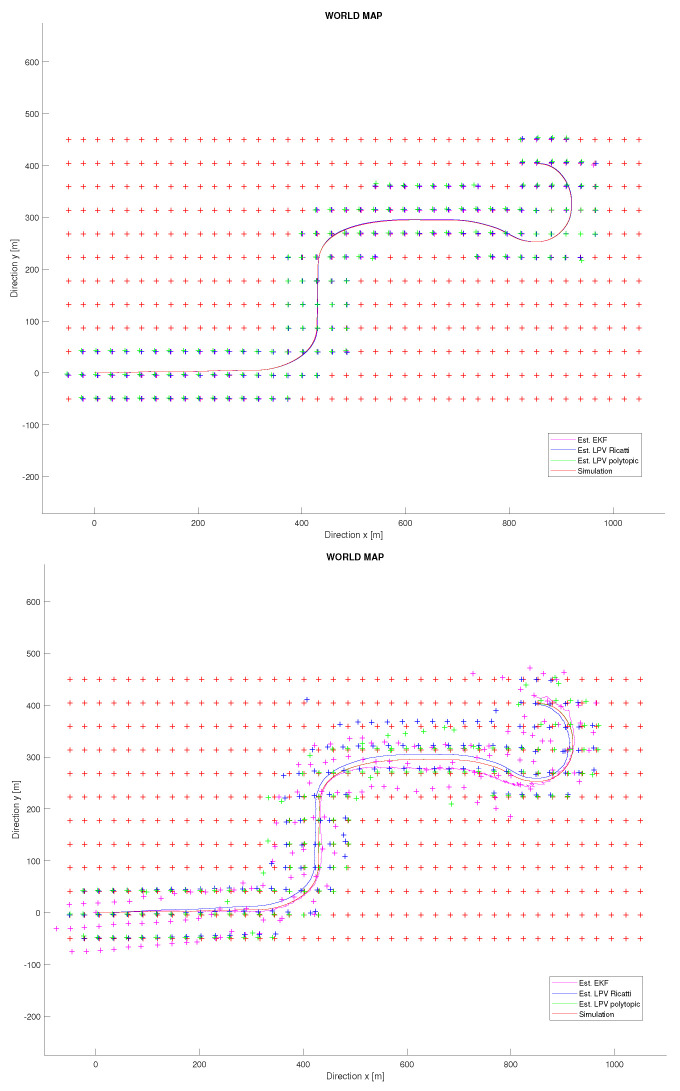
Vehicle trajectory at the world map. **Up**: Simulation with noisy initialization of landmarks. **Down**: Simulation with zero initialization of landmarks.

Figure 7, left, shows the results of the mapping task for this experiment. It can be seen how, unless using a sensor with bad accuracy for the initialization of landmarks, both proposed techniques are robust enough to converge close to the real landmark’s position. Furthermore, note how the vehicle discovers its closest surroundings and accurately places the landmarks in the incremental map. At the same time, the vehicle trajectory on the map can also be determined correctly. Therefore, the mapping task is carried out properly for both techniques, and no significant differences are observed.

**Figure 8:**
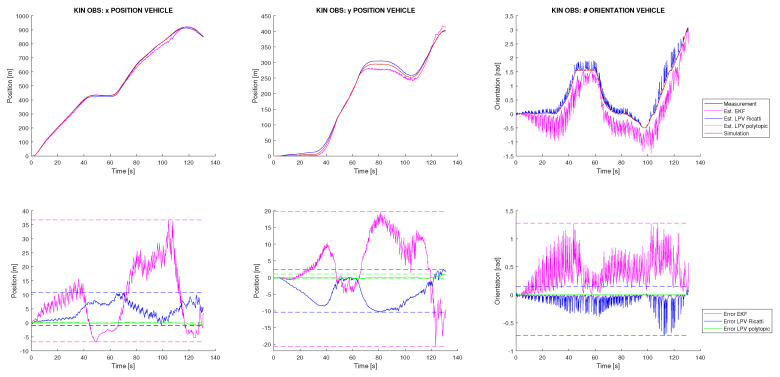
Vehicle kinematic state estimation from the simulation with friction, Gaussian noise and zero initialization of landmarks.

The second experiment belongs to a critical case in which no state initialization mechanism is available. The EKF-SLAM technique is very sensitive if new discovered landmarks are not properly initialized. In [20], the initialization process for the SLAM system state vector $\vec{x}$ and the SLAM system variance matrix *P* is described. This process was applied in the previous experiment, but now it is removed and all landmarks initialization are set to nearly zero. This will allow us to more deeply analyse the robustness of the proposed SLAM solution and check if this initialization is needed for the proposed methodologies. Figure 8 shows the kinematic results for this experiment. It is seen how, as expected, the EKF is not able to deal with this high uncertainty. Similarly, the Riccati observer also cannot assume it, but, on the contrary, the polytopic approach is able to perform a good localization with an error of decimetres for the vehicle position. These results show how this technique outperforms the robustness of the classical EKF when no accuracy on landmark initialization is able. Furthermore, Figure 7, right, shows the results of the mapping task for this experiment. It can be observed how only the polytopic observer is able to converge and accurately place the discovered landmarks in the incremental map. For the other techniques, a significant drift on the vehicle trajectory is appreciated, and in particular for the EKF, significant oscillations appear in every landmark discovery.

In conclusion, the presented results show how the the dynamic estimation tasks are developed correctly for both proposed techniques, apparently without significant differences at this level. However, achieving accuracy in the vehicle location becomes more complex, and the best estimation is provided by the polytopic observer. The Ricatti observer is conceived as the direct evolution of the EKF-SLAM methodology to include the dynamic response of the vehicle and use LPV techniques. It is seen how this approximation fails, and the results become worse with respect to the classical EKF. For this reason, it is necessary to go in depth into the LPV techniques and find a new approximation of the SLAM problem based on the polytopic models and tuning methodologies based on the optimization of a set of LMIs. This approximation turns out to be more accurate because it avoids updating the system model at the operation point, performing an interpolation for the whole problem domain. This weighing allows Lyapunov stability conditions to be accomplished for the global system and allows one to reach a better observation of the nonlinear problem. This is the improvement to the SLAM problem introduced in this article.

## 6. Conclusions

In this article, the SLAM problem is solved by applying a methodology based on kinematic/dynamic models using the LPV framework. The proposed SLAM approach is designed for mobile platforms with a considerable mass and not operated at low velocities, such as autonomous vehicles. For this type of mobile platform, its dynamic response becomes evident and contrasts with the mobile robots considered in classical SLAM problems, where only kinematic effects are modelled. A difference from those classical approaches is that in this proposal, an estimation mechanism of the dynamic response of the vehicle is provided, which is combined with the solution to a SLAM problem. Furthermore, this approach proves that to solve a problem with important nonlinear behaviour, it is necessary to go in depth into advanced control techniques, avoiding the classical EKF, which linearizes the system model around the operation point. The approach introduced in this paper is based on the use of the LPV technique, which considers the whole problem domain and avoids any linearization, and it has been verified in simulation, showing the advantages of the polytopic stationary observer with respect to the Ricatti iterative observer. To implement the polytopic estimator, a reformulation of the SLAM problem has been necessary, developing the map kinematic model presented in Equation (5), which allows a linear observation model for the SLAM problem and introduces a robocentric point of view to it. Furthermore, the polytopic technique only requires considering the measurable surroundings of the vehicle, avoiding the maintenance of the whole discovered map. It is verified in simulation that these relaxations do not have adverse effects to the localization task and allows it outperform the classical EKF-based methods.

Moreover, the proposed approach considers an observation system with an architecture that couples with the kinematic/dynamic controller structured in layers for an autonomous vehicle proposed at [2]. Both systems are built using the same vehicle models and are implemented with gain-scheduling LPV techniques. Both systems allow showing that it is possible to separate the kinematic and dynamic responses, permitting perform the dynamic treatment independently to the kinematic treatment. At the same time, in this article is shown how the vehicle kinematic estiamted layer can be combined with a SLAM problem, in the same way as in [3] the vehicle kinematic control layer is combined with the trajectory planner. Therefore, the presented kinematic/dynamic localization system allows to advance in the development of a layered software which permits the autonomous driving of a vehicle.

As future work, the following aspects will be further researched:

- The proposed approach will be applied and tested in the real vehicle in the context of current ongoing research project.
- The extension of the proposed observation approach to take into account 3D scene factors (such as ground unevenness and vehicle instability) will be explored.
- The inclusion of range sensors instead of state sensors will also be considered.

## Figures and Tables

**Table 1:** Dynamic model parameters of Tazzari Zero vehicle, which belongs to the Elektra research project [15].

Dynamic Model Parameters of Tazzari Zero Vehicle		
**Par.**	**Description**	**Value**
*a*	Distance from CoG to front shaft	0.758 m
*b*	Distance from CoG to rear shaft	1.036 m
*m*	Vehicle mass	683 kg
*I*	Vehicle inertia	561 kg·$m^{2}$
$C_{x}$	Tire stiffness coefficient	15,000 $\frac{N}{\mathrm{rad}}$
$C_{d}$	Vehicle drag coefficient	0.5
*A*	Vehicle frontal area	4 m${}^{2}$
ρ	Air density at 25 °C	1.2 $\frac{\mathrm{kg}}{m^{3}}$
μ	Friction coefficient tire-ground	variable

**Table 2:** Installation parameters of the exteroceptive sensor on the Tazzari Zero vehicle.

Installation Parameters of LIDAR Sensor		
**Par.**	**Description**	**Value**
*s*	Distance from CoG to sensor on the vehicle longitudinal direction	0.3 m
*t*	Distance from CoG to sensor on the vehicle cross direction	0.1 m
β	Sensor orientation respect to the vehicle longitudinal direction	90°

## Data Availability

All the required data is included in the paper.
